# Successful management of scrub typhus-associated hemophagocytic lymphohistiocytosis with doxycycline and immunomodulation: a case report

**DOI:** 10.3389/fped.2026.1787726

**Published:** 2026-04-29

**Authors:** Yuanyuan Hua, Yan Zhang, Jimin Zhou, Yan Shi, Feng Xu

**Affiliations:** 1 Department of Pediatrics, the First People’s Hospital, Liangshan Yi Autonomous Prefecture, Sichuan, China; 2 Department of Intensive Care Unit, Children’s Hospital of Chongqing Medical University, National Clinical Research Center for Children and Adolescents’ Health and Diseases, Ministry of Education Key Laboratory of Child Development and Disorders, Chongqing Key Laboratory of Pediatric Metabolism and Inflammatory Diseases, Chongqing, China; 3 Department of Pediatrics, Jin Yang County People’s Hospital, Liangshan Yi Autonomous Prefecture, Sichuan, China

**Keywords:** doxycycline, hemophagocytic lymphohistiocytosis, immunomodulation, orientia tsutsugamushi, scrub typhus

## Abstract

**Introduction:**

Hemophagocytic lymphohistiocytosis (HLH) is a fatal hyperinflammatory complication of scrub typhus, often with delayed diagnosis. This case report aims to detail the clinical trajectory and successful management of scrub typhus-associated HLH in a child, highlighting key diagnostic clues.

**Case presentation:**

A previously healthy 14-year-old boy from a scrub typhus-endemic area presented with prolonged high fever, cough, and lethargy. Physical examination revealed a pathognomonic eschar.

**Results:**

Laboratory findings fulfilled HLH-2004 diagnostic criteria, including cytopenias, hyperferritinemia (>3,000 ng/mL), hypofibrinogenemia, and hemophagocytosis on bone marrow aspirate. The patient was diagnosed with secondary HLH triggered by scrub typhus. Immediate treatment with oral doxycycline, combined with immunomodulation using intravenous immunoglobulin and dexamethasone, led to fever resolution within 72 h and full recovery.

**Conclusion:**

This case highlights that in pediatric scrub typhus, a rapid decline in platelets/fibrinogen and a sharp rise in ferritin/LDH should alert clinicians to possible HLH. Early combined therapy targeting both the infection (e.g., doxycycline) and the cytokine storm (e.g., immunomodulators) may be beneficial for favorable outcomes.

## 1 Introduction

Hemophagocytic lymphohistiocytosis (HLH) is a life-threatening hyperinflammatory syndrome characterized by a dysregulated immune response (1). In children, HLH is most frequently triggered by infections. Among these, scrub typhus—a mite-borne zoonosis caused by Orientia tsutsugamushi—has been increasingly recognized as a significant etiological agent in endemic regions of the Asia-Pacific “tsutsugamushi triangle” (2–4).

The diagnostic challenge of scrub typhus-associated HLH is well-documented. A significant body of literature, including series analyses of pediatric cases, has elucidated its high rate of initial misdiagnosis—often as sepsis or severe infection—due to non-specific presentations and frequent oversight of the pathognomonic eschar (5, 6). These studies have crucially emphasized meticulous physical examination as the first step to avoid diagnostic delay (5, 6).

This diagnostic difficulty is of particular concern in China, where the incidence of scrub typhus has risen, with hyperendemic areas including Liangshan Prefecture in Sichuan Province (7, 8). Despite awareness, early recognition of complicating HLH remains challenging in clinical practice, as its insidious onset mimics severe infection, often leading to delayed diagnosis of this fatal complication, including disseminated intravascular coagulation and multiorgan failure, even in adults (9).

Herein, we report a detailed case of scrub typhus-associated HLH in a child from this endemic region. Through this case and a review of the clinical features, we aim to highlight key diagnostic clues and therapeutic considerations to aid in more timely diagnosis and intervention.

## 2 Case presentation

A previously healthy 14-year-old boy from a rural area in Liangshan Prefecture was transferred to our hospital with a 6-day history of cough, fever (up to 39.5 °C), and progressive lethargy. He had a history of recent outdoor activity.

### 2.1 Physical examination

On admission, his vital signs were: temperature 39.2 °C, heart rate 122 bpm, respiratory rate 38/min, blood pressure 108/64 mmHg, and oxygen saturation 96% on room air. He appeared acutely ill and lethargic but responsive.

**Figure 1:**
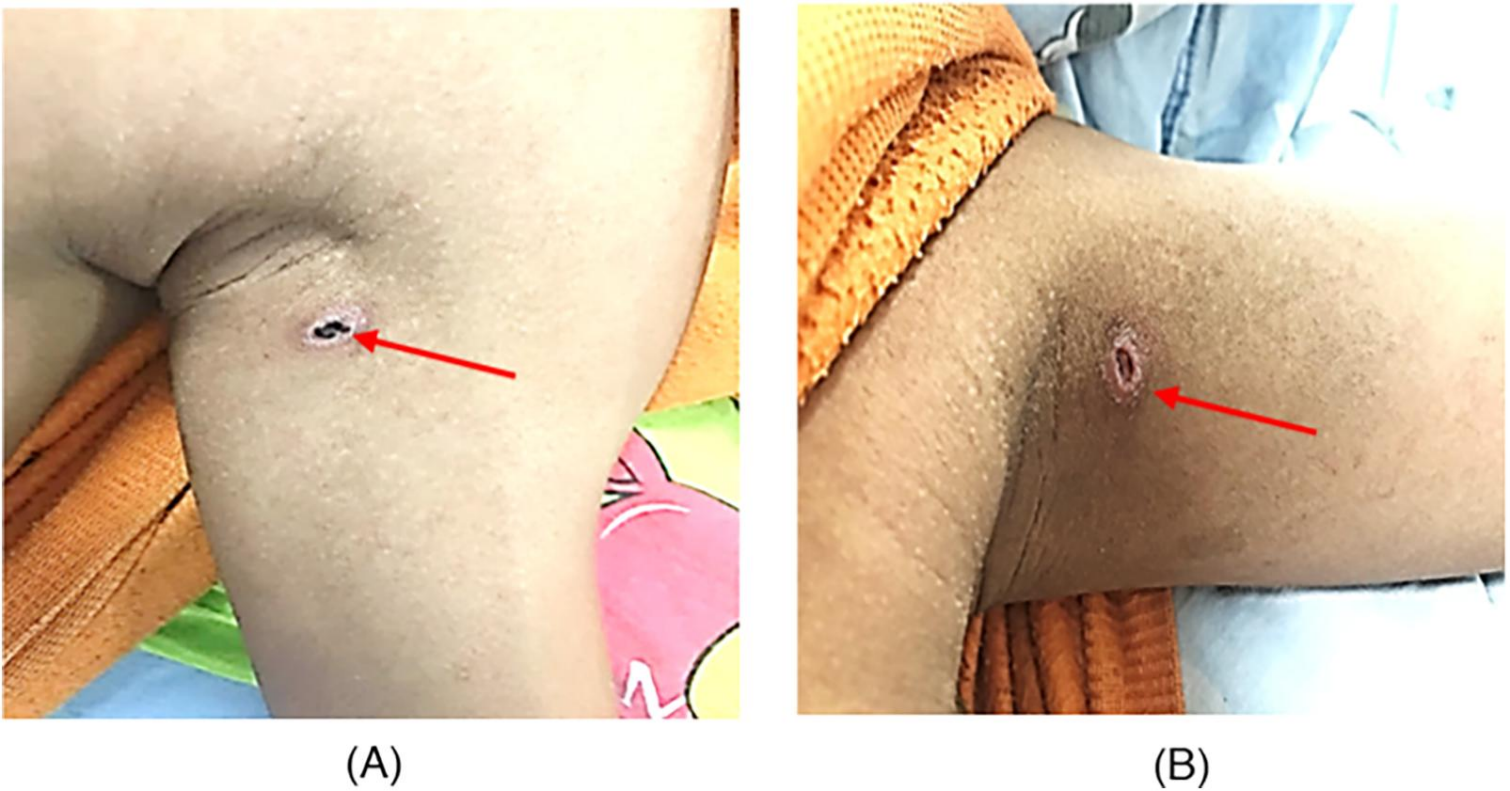
Clinical evolution of the pathognomonic eschar in a 14-year-old male patient with scrub typhus-associated hemophagocytic lymphohistiocytosis. **(A)** Black eschar (red arrow) surrounded by an erythematous halo located in the left axilla on the day of admission (day 0). **(B)** Crater-like ulceration (red arrow) observed after the eschar spontaneously sloughed off on day 3 of hospitalization.

Skin: A pathognomonic black eschar surrounded by an erythematous halo was identified in the left axilla (Figure 1A). No jaundice, pallor, skin rashes, or petechiae were observed. Three days later, the eschar sloughed off, leaving a crater-like ulceration (Figure 1B).

Lymph nodes: multiple small (approximately 0.5–1 cm), non-tender lymph nodes became palpable in the cervical and inguinal regions.

Head and neck: Tonsils were not enlarged. The neck was supple with no meningeal signs.

Chest and cardiovascular: Chest expansion was symmetrical with clear breath sounds bilaterally; no wheezes or crackles were auscultated. The heart rate was regular with no murmurs, rubs, or gallops.

Abdomen: The abdomen was soft and non-distended, with mild periumbilical tenderness but no guarding or rebound. The liver was not palpable. Splenomegaly was present, with the spleen palpable 3.5 cm below the left costal margin. No abdominal masses were detected. Bowel sounds were normal.

Extremities and neurological: No clubbing, cyanosis, or edema was present. Peripheral perfusion was adequate with capillary refill time <3 s. The patient was lethargic but oriented. Cranial nerves were intact. Motor strength was 5/5 in all extremities. Deep tendon reflexes were normal. Plantar responses were flexor. No focal neurological deficits or meningeal signs were identified.

### 2.2 Laboratory and imaging findings

**Figure 2:**
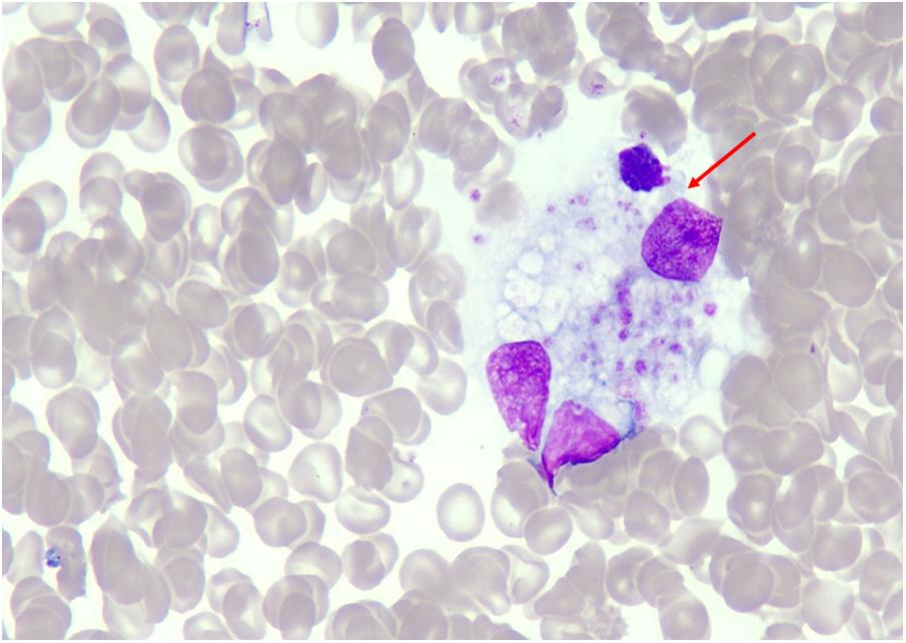
Bone marrow aspirate cytology showing hemophagocytosis in a 14-year-old male patient with scrub typhus-associated hemophagocytic lymphohistiocytosis. Wright-Giemsa stain, ×1,000 oil immersion objective. A histiocyte in the center of the image (red arrow) is seen engulfing multiple platelets. This finding fulfills one of the HLH-2004 diagnostic criteria. The bone marrow aspirate was obtained on day 0 (admission day).

Notable laboratory results are summarized in Table 1. Key findings included severe thrombocytopenia, leukopenia, hypoalbuminemia, and hyponatremia. Marked elevations in lactate dehydrogenase (LDH), ferritin, triglycerides, D-dimer, and liver transaminases were observed. Fibrinogen was low. Bone marrow cytology revealed histiocytes engulfing platelets (Figure 2). Chest CT showed bilateral pulmonary infiltrates, mediastinal/axillary lymphadenopathy, and splenomegaly.

**Table 1:** Key laboratory findings of the index patient on admission (day 0) compared with reference ranges.

Parameter	Patients’ value	Unit	Reference range
Complete blood count			
White blood cell count (WBC)	1.83	×10^9^/L	4.0–10.0
Absolute neutrophil count (ANC)	0.87	×10^9^/L	1.80–6.30
Platelet count	20	×10^9^/L	100–300
Hemoglobin	116	g/L	120–160
Biochemistry & electrolytes			
Albumin	32	g/L	35–55
Alanine aminotransferase (ALT)	73	U/L	<40
Aspartate aminotransferase (AST)	180	U/L	<40
Lactate dehydrogenase (LDH)	776	U/L	120–250
Sodium	120.6	mmol/L	135–145
Urea	10.3	mmol/L	2.8–7.2
Creatinine	137	μmol/L	44–133
Inflammation & coagulation			
C-reactive protein (CRP)	79.40	mg/L	<8
Ferritin	>3,000	ng/mL	30–400
Triglycerides	3.19	mmol/L	<1.70
Fibrinogen	0.890	g/L	2.0–4.0
D-dimer	19.57	mg/L FEU	<0.5

Reference ranges are according to the clinical laboratory standards of The First People's Hospital of Liangshan Prefecture. WBC, white blood cell count; ANC, absolute neutrophil count; ALT, alanine aminotransferase; AST, aspartate aminotransferase; LDH, lactate dehydrogenase; CRP, C-reactive protein; FEU, fibrinogen equivalent units.

### 2.3 Diagnosis and treatment

The patient met 7 out of 8 HLH-2004 diagnostic criteria (10): fever, splenomegaly, cytopenias affecting ≥2 lineages, hypertriglyceridemia/hypofibrinogenemia, hyperferritinemia, and hemophagocytosis in bone marrow. Combined with the epidemiological context and the pathognomonic eschar, a diagnosis of scrub typhus-induced secondary HLH was made.

Management consisted of immediate oral doxycycline (4 mg/kg/day), which was continued for a total of 14 days. For HLH, immunomodulation with intravenous immunoglobulin (IVIG) at 1 g/kg/day for 2 consecutive days and dexamethasone at 10 mg/m^2^/day was initiated (11). Dexamethasone was administered at this dose for 7 days, then tapered over the following 7 days prior to discharge. Supportive care included infusion of human fibrinogen and albumin. His fever subsided within 72 h, followed by gradual normalization of laboratory parameters. The clinical course and key interventions are summarized in Table 2.

**Table 2:** Timeline of clinical events and therapeutic interventions during hospitalization for a 14-year-old male patient with scrub typhus-associated hemophagocytic lymphohistiocytosis.

Hospital day	Event
Day 0	Admission; scrub typhus diagnosed; doxycycline started; bone marrow aspiration performed
Day 2	Bone marrow confirmed hemophagocytosis; HLH diagnosed; dexamethasone + IVIG initiated
Day 3	Fever peak decreased
Day 4	Fever resolved; blood counts improved (platelets 93 × 10^9^/L, WBC 6.88 × 10^9^/L, ANC 3.35 × 10^9^/L)
Day 7	Splenomegaly and lymphadenopathy resolved
Day 15	Laboratory parameters normalized except for mild ALT elevation (147 U/L)
Day 16	Discharged

Day 0 corresponds to the day of admission. During hospitalization, the patient received hepatoprotective agents, albumin, and fibrinogen supplementation. HLH, hemophagocytic lymphohistiocytosis; IVIG, intravenous immunoglobulin; WBC, white blood cell count; ANC, absolute neutrophil count; ALT, alanine aminotransferase.

### 2.4 Follow-up and outcomes

At discharge, the patient was afebrile and asymptomatic, with no palpable splenomegaly or lymphadenopathy. Laboratory parameters were largely normalized except for mild elevation of alanine aminotransferase (ALT 147 U/L). He was prescribed oral polyene phosphatidylcholine capsules for hepatoprotection and advised to continue follow-up at the hematology clinic. Telephone follow-up after discharge revealed that liver function tests had normalized and the patient remained asymptomatic, with no recurrence of HLH symptoms. No adverse events or drug intolerance were reported during hospitalization.

### 2.5 Patient perspective

The patient's father shared the family's experience. Initially, they did not realize the severity of the illness and felt extremely worried when the diagnosis was explained and the critical condition notice was issued. After treatment began, they observed the child's rapid improvement, which gradually gave them confidence. Regarding treatment choices, they initially had concerns about using doxycycline and dexamethasone in a child but understood the necessity after the physician's explanation and agreed to the regimen. The father expressed satisfaction with the outcome, noting that the child had fully recovered and returned to normal life. Throughout hospitalization, they trusted the medical team's decisions.

## 3 Discussion

The documented overall mortality rate of scrub typhus-associated HLH was 6.7% (12). Early diagnosis is paramount, as delayed treatment leads to high mortality.

Our case and literature review highlight several key clinical clues for early suspicion of HLH in scrub typhus patients: (1) Prolonged high-grade fever (>7 days) refractory to initial management (13); (2) Rapidly progressive cytopenias, especially thrombocytopenia, which is often the earliest and most dramatic sign (13–15); (3) Profound hypoalbuminemia and sharply rising LDH, indicative of severe systemic inflammation and cell damage (15); (4) Hyperferritinemia at orders of magnitude above typical infection levels (15, 16).

The characteristic eschar is the diagnostic cornerstone for scrub typhus but can be missed in up to 50% of cases, particularly in severe illness where thorough examination is challenging or if located in occult areas (17, 18). Therefore, in endemic regions, any child with unexplained febrile illness with multiorgan involvement should be meticulously examined for an eschar.

It should be noted that hemophagocytosis on bone marrow examination is not specific for HLH and can be observed in various severe infections, including scrub typhus itself, reflecting a state of marked immune activation. In this case, the bone marrow finding was considered as one component of the HLH-2004 criteria, and the diagnosis was established based on the fulfillment of multiple criteria rather than this finding alone.

Differential diagnoses were carefully considered. Based on the initial presentation of fever, cytopenias, and multiorgan dysfunction, severe sepsis was the primary consideration; however, the clinical and laboratory features of sepsis and HLH overlap significantly, and studies have shown that a subset of patients with severe sepsis or septic shock fulfill HLH diagnostic criteria, which is associated with higher mortality (19). From an immunological perspective, HLH and sepsis exhibit distinct T-cell activation profiles: HLH is characterized by expansion of CD38high/HLA-DR + CD8+ T cells, reflecting robust T-cell activation and interferon-γ-driven inflammation, whereas this phenotype is notably absent in patients with early sepsis (20, 21). These findings suggest that, despite overlapping clinical features, the underlying pathophysiology of HLH and sepsis differs fundamentally, with T-cell hyperactivation playing a central role in HLH. Although flow cytometric assessment of T-cell activation was not performed in our patient, the above evidence suggests that immunophenotyping may inform future differentiation between HLH and sepsis, particularly in resource-limited settings where timely diagnosis is critical.

In the present case, the presence of a pathognomonic eschar pointed toward scrub typhus as the underlying trigger. Epstein–Barr virus (EBV)-associated HLH was considered, as EBV is a common cause of secondary HLH in children (4, 22), but was ruled out by negative EBV serology. Other viral infections (influenza, adenovirus, etc.) are also recognized triggers of HLH (22) and were excluded by negative testing. Malignancy, such as leukemia or lymphoma, can also trigger HLH (22) but was considered unlikely due to the absence of blasts on peripheral smear and the presence of a clear infectious trigger. Familial HLH typically presents in infancy or early childhood; in a cohort of 122 children who met HLH-2004 diagnostic criteria, Chinn et al. (23) found that all patients with biallelic familial HLH gene defects presented before 1 year of age, and only 7% of patients aged 12–18 years had an identifiable genetic etiology. In the present case, the patient's age (14 years), absence of family history of HLH or unexplained childhood deaths, and rapid response to targeted therapy all argued against familial HLH, although genetic testing was not performed due to financial constraints and an underlying genetic predisposition cannot be entirely excluded.

For children with severe scrub typhus complicated by HLH, the treatment paradigm must address both the underlying infection and the consequent hyperinflammatory storm. Prompt and effective anti-infective therapy is the cornerstone. For the anti-infective treatment of severe scrub typhus in children, high-level evidence specific to the pediatric population is currently lacking, although a large-scale trial in adults supports combination therapy (24). A recent systematic review suggested that doxycycline might lead to faster defervescence than azithromycin in severe cases (25). This potential for a rapid clinical response guided our choice of doxycycline as the foundational anti-infective agent, which was followed by the patient's swift defervescence.

Concurrently, timely immunomodulation is critical to abate the cytokine storm. Our strategy of combining intravenous immunoglobulin (IVIG) with dexamethasone aligns with a stepwise approach advocated for managing infection-triggered secondary HLH. A real-world study from a developing country, where infections constituted the majority of HLH triggers, implemented a protocol in which IVIG serves as the first-line immunomodulatory agent for cases with a clear infectious etiology (26). This approach prioritizes controlling hyperinflammation while mitigating the risk of profound immunosuppression that could exacerbate the underlying infection. Notably, a report specifically addressing scrub typhus-associated HLH has proposed that a combination of doxycycline, IVIG, and glucocorticoids may constitute an effective therapeutic regimen (27).

The favorable outcome in our case illustrates the principle of etiology-specific management in secondary HLH. The prognosis of HLH is highly dependent on its trigger. A systematic review of critically ill children with HLH indicated that outcomes for secondary HLH are generally more favorable than those for primary HLH when the trigger is effectively controlled (28). By promptly addressing the scrub typhus infection and concurrently modulating the immune response, we successfully interrupted the vicious cycle of infection-driven HLH. This aligns with the observation that scrub typhus-associated HLH carries a more favorable prognosis with appropriate treatment compared to other causes of secondary HLH (12).

It is noteworthy that while the individual components of our regimen (doxycycline, corticosteroids, IVIG) are well-established in their respective domains, literature specifically evaluating their combination for scrub typhus-associated HLH remains limited (6). This gap underscores the need for more clinical reports to consolidate evidence. Our detailed case provides a descriptive account of one successful outcome with this combination therapy. Further studies are needed to validate its effectiveness in similar clinical scenarios.

This report has several limitations. First, laboratory confirmation of scrub typhus (e.g., specific antibody testing or PCR) was not available due to resource constraints at the treating facility; the diagnosis was therefore based on characteristic clinical features (fever and eschar) and epidemiological history, consistent with the clinical diagnostic criteria of the Chinese Expert Consensus on the Clinical Diagnosis and Treatment of Scrub Typhus (2024) (29). Second, genetic testing for familial HLH was not performed due to financial constraints. Although the patient's age (14 years), clear infectious trigger, negative family history, and rapid treatment response all support secondary HLH, the possibility of an underlying genetic predisposition cannot be entirely excluded. Despite these limitations, the clinical presentation and treatment outcome of this case provide valuable insights for clinicians in similar resource-limited settings.

## 4 Conclusion

Pediatricians in scrub typhus-endemic areas must maintain a high index of suspicion for secondary HLH. Beyond the established HLH-2004 criteria, vigilant monitoring for dynamic warning signs—such as rapidly declining albumin, escalating lactate dehydrogenase, and new-onset respiratory or neurological symptoms—can facilitate earlier detection. This case suggests that early dual-pathway therapy—combining timely, targeted antibiotics (e.g., doxycycline) with immunomodulators (e.g., corticosteroids with or without intravenous immunoglobulin)—may be beneficial in managing scrub typhus-associated HLH. However, as this is a single retrospective case report, these findings should be interpreted descriptively, and further research is needed to confirm the effectiveness of this approach. With heightened awareness, timely diagnosis and treatment of this life-threatening complication may lead to favorable outcomes.

## Data Availability

The raw data supporting the conclusions of this article will be made available by the authors, without undue reservation.
